# Efficacy of Systemic Amoxicillin–Metronidazole in Periodontitis Patients with Diabetes Mellitus: A Systematic Review of Randomized Clinical Trials

**DOI:** 10.3390/medicina58111605

**Published:** 2022-11-07

**Authors:** Maryam Hassan Mugri

**Affiliations:** Department of Maxillofacial Surgery and Diagnostic Sciences, College of Dentistry, Jazan University, Jazan 45142, Saudi Arabia; mmugri@jazanu.edu.sa or dr.mugri@gmail.com; Tel.: +966-504654682

**Keywords:** periodontitis, diabetes mellitus, systemic antibiotics, amoxicillin, metronidazole, non-surgical therapy

## Abstract

Systemic amoxicillin–metronidazole was proven to be effective in managing periodontitis in systemically healthy patients. It was demonstrated that systemic antibiotic therapy can effectively improve clinical periodontal parameters and reduce periodontopathogenic organisms in the subgingival biofilm. However, the evidence for prescribing this drug combination to patients with diabetes remains insufficient. This systematic review was designed to evaluate the effectiveness of a systemic amoxicillin–metronidazole combination as an adjunct to nonsurgical periodontal therapy in patients with diabetes presenting with chronic periodontitis. The PubMed, Scopus, and Web of Science databases were electronically searched for randomized clinical trials in January 2022. Randomized clinical trials evaluating systemic amoxicillin–metronidazole therapy as an adjunct to nonsurgical periodontal therapy in patients with type 2 diabetes presenting with periodontitis were selected for screening. The qualities of the studies were assessed using the Cochrane Collaboration’s Tool for Assessing Risk of Bias Version 2.0 (ROB-2), and a GRADE assessment was applied to estimate the overall certainty of the evidence. Using predefined eligibility criteria, four clinical trials examining 209 patients were selected from the 611 articles identified in the search. Two studies reported a better reduction in clinical parameters when SRP was combined with systemic amoxicillin–metronidazole. Systemic amoxicillin–metronidazole was found to be as effective as clindamycin. Surgical therapy with systemic amoxicillin–metronidazole was more effective than nonsurgical therapy with systemic amoxicillin–metronidazole, even though both resulted in reduced clinical parameters. Combined amoxicillin–metronidazole was observed to reduce periodontal probing depth (PPD), clinical attachment level (CAL), and bleeding on probing (BOP) compared to no treatment or NSPT alone. However, the effect was not greater when compared to NSPT with clindamycin or surgical therapy with amoxicillin–metronidazole. Further randomized trials are required before clinical guidelines can be established for the use of systemic amoxicillin–metronidazole. Future randomized controlled clinical trials with long-term follow-ups are required to assess the efficacy of systemic antibiotic therapy in managing periodontitis in patients with diabetes.

## 1. Introduction

Diabetes mellitus is a chronic, non-communicable, metabolic disease characterized by hyperglycemia and is attributed to a combination of defective insulin secretion by β-cells of the pancreas and the inability of insulin-sensitive tissues to respond to the secreted insulin [1]. Chronic hyperglycemia can lead to several complications, including cardiovascular diseases, nephropathy, and retinopathy, and is associated with severe morbidity and mortality. Diabetes affects more than 537 million adults globally, which represents over 10.5% of the population. This number is expected to rise to 786 million over the next two decades [2]. The International Diabetes Federation (IDF) estimates that diabetes mellitus and associated complications caused 6.7 million deaths during 2021 alone [3]. This disease is also the ninth major cause of reduced life expectancy [4,5,6].

The American Diabetes Association described periodontal disease as the sixth major complication of diabetes mellitus [7]. Diabetes and periodontitis are comorbid conditions [8], with diabetes considered a risk factor that modifies the grade of periodontitis in the current periodontal disease classification [9,10]. Additionally, a bidirectional relationship exists between periodontitis and diabetes mellitus [11,12].

With the increasing prevalence of type 2 diabetes mellitus and periodontitis, it is necessary to systematically and critically re-evaluate treatment strategies for patients with diabetes presenting with periodontitis. In patients with diabetes, the treatment of periodontitis is primarily aimed at removing plaque and biofilm [13]. Biofilm control is achieved through professional mechanical plaque removal via scaling and root planing with adjuvant patient-administered plaque control measures, irrespective of the glycemic status of the patient. It is vital to evaluate any associated complications before formulating a treatment plan. In patients with poorly controlled diabetes mellitus, glycemic levels must be controlled before instituting any surgical or invasive periodontal procedures. Patients should be kept on a strict maintenance protocol until success can be achieved [12]. Concurrent management of diabetes mellitus is required to achieve optimal periodontal health.

While nonsurgical periodontal therapy (NSPT) can reduce the clinical and inflammatory parameters in patients with poorly controlled diabetes, adjunctive use of systemic antibiotics may improve the clinical outcomes [14,15]. Amoxicillin when combined with metronidazole has been demonstrated to have synergistic action, thus reducing the required dosage of both drugs for biological action [16]. Zandbergen et al. reported an improvement in clinical outcomes in systemically healthy chronic periodontitis patients when an amoxicillin–metronidazole combination was used as an adjunct to standard NSPT. However, the evidence regarding its efficacy in systemically compromised individuals, such as patients with diabetes mellitus, is contradictory and requires more investigation before the formulation of clinical guidelines [17]. Santos et al. published a systematic review assessing the efficacy of systemic antibiotics as adjuncts to NSPT in diabetic patients. Of the five included articles, three studied doxycycline, one investigated azithromycin and one study assessed an amoxicillin–metronidazole combination. Overall, the authors concluded that systemic antibiotics provided a small improvement in clinical parameters [18]. In another systematic review, Souto et al. concluded that an amoxicillin–metronidazole combination provided a better reduction in probing depth than doxycycline, azithromycin, or amoxicillin. However, only one of the studies included was of an amoxicillin–metronidazole combination [19]. The systematic reviews published to date have evaluated all systemic antibiotics used in conjunction with NSPT in patients with diabetes and these reviews only include one single trial evaluating an amoxicillin–metronidazole combination. With Ong et al. reporting the amoxicillin–metronidazole combination to be one of the most prescribed antibiotics by periodontists and as the adverse effects associated with long-term and unnecessary antibiotic use, including the emergence of antibiotic-resistant organisms, increase globally, it has become increasingly critical to evaluate whether the prescription of antibiotics helps in the improvement of clinical outcomes in inflammatory periodontal diseases [20,21,22]. This systematic review is aimed at evaluating the current evidence on the efficacy of an amoxicillin–metronidazole combination in improving periodontal parameters in patients with type 2 diabetes mellitus compared to standard periodontal therapy.

## 2. Methodology

### 2.1. Search Criteria

Preferred Reporting for Systematic Reviews and Meta-analysis (PRISMA) guidelines 2020 were followed in the design and execution of the systematic review [23].

The focused question was as follows: “Does administration of a systemic amoxicillin–metronidazole combination after nonsurgical periodontal therapy in diabetic patients presenting with periodontitis result in better clinical treatment outcomes compared to those who received nonsurgical periodontal therapy alone?”

The inclusion criteria according to PICOS were as follows:

Population (P): diabetic patients presenting with periodontitis;

Intervention (I): systemic amoxicillin–metronidazole combination after nonsurgical periodontal therapy;

Comparison (C): nonsurgical periodontal therapy alone, or in combination with systemic antibiotics other than amoxicillin–metronidazole;

Outcome (O): pocket probing depth (primary outcome parameter) and/or reduced bleeding on probing and clinical attachment level (secondary outcome parameters);

Studies (S): randomized clinical trials and clinical control trials with a follow-up of ≥3 months.

A study protocol was prepared to identify randomized controlled trials conducted among periodontitis patients with type 2 diabetes mellitus treated with nonsurgical periodontal therapy in combination with systemic amoxicillin–metronidazole compared to those treated with nonsurgical periodontal therapy alone or in combination with other systemic antibiotics. A reduction in pocket probing depth was the primary outcome parameter, while bleeding on probing (BOP) and clinical attachment loss (CAL) were secondary outcome parameters. The study protocol was registered in Prospero under the ID CRD42022321036.

### 2.2. Screening and Selection of Studies

The electronic databases of MEDLINE (PubMed), Scopus, and Web of Science were searched for relevant articles in January 2022. Articles in the English language with full-text digital copies were considered. The search strategy used a combination of the following keywords: periodontitis, systemic antibiotics, amoxicillin, metronidazole, and nonsurgical therapy. The search strategy is described in detail in Supplementary Table S1.

Two reviewers (MM, MAK) independently screened the titles and abstracts of the search results. After deleting duplicate articles, the resulting studies were reviewed against the inclusion criteria. The full texts of all studies of possible relevance were obtained for assessment against the stated inclusion criteria. Only studies that fulfilled the criteria were further assessed to synthesize the results. The reference list of the included articles was assessed for any studies that fulfilled the inclusion criteria.

### 2.3. Extraction of Data

Customized tables were designed in Microsoft Excel (Microsoft Inc., Redwood, CA, USA) for data extraction. Two reviewers (MM, MAK) independently extracted relevant data from the selected studies, including authorship and year of publication, sample characteristics, study design, outcomes, and inferences. Any disagreements were resolved through discussion with a third reviewer (AB). Additional parameters, including microbiological parameters, biomarker levels, tooth mobility, number of participants in each selected study, and any reported adverse effects, were also assessed and entered into the data extraction table.

### 2.4. Assessment of Risk of Bias in Selected Studies

The risk of bias was assessed by two authors independently (MM, MAK) using the Cochrane Collaboration’s Tool for Assessing Risk of Bias Version 2.0 (ROB 2 tool) [24]. Any disagreements in the ratings were resolved through discussion with a third reviewer (AB). The validity of the data was evaluated against five specific domains: bias due to randomization, deviation from intended intervention, missing outcome data, bias in the measurement of the outcome, and bias in the selection of the reported result and was assessed as low bias, some concerns, and high bias.

### 2.5. Quality of Evidence for Outcomes in the Summary of Findings Table

GRADE guidelines were applied to evaluate the overall certainty of evidence for the outcomes mentioned in the summary of findings table [25]. One author (MM) applied the GRADE system, and the final rating was decided after discussion with the two other reviewers (MAK, AB). The risk of bias, inconsistency of results, indirectness of evidence, imprecision of results, and publication bias were evaluated and rated. Initially, the evidence for primary outcome was rated as high and then downgraded if any serious concerns were detected regarding the risk of bias, inconsistency in the reporting of the results of the primary outcomes, indirectness of evidence, imprecision in the results, or publication bias.

## 3. Results

**Figure 1:**
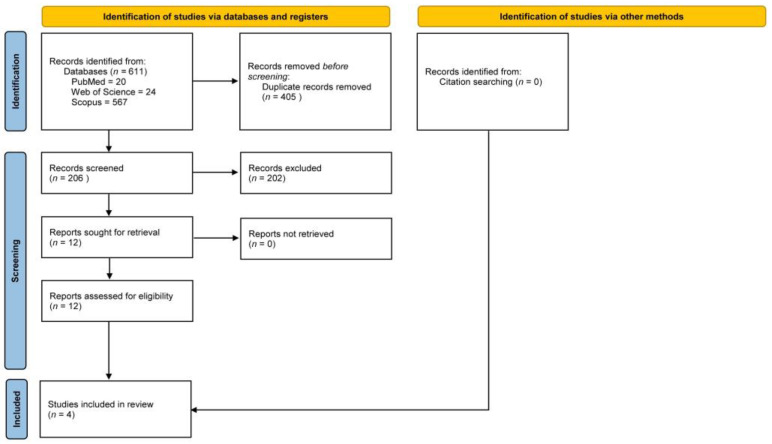
PRISMA flow diagram of the review.

The electronic initial search of the databases yielded 611 articles. In total, 405 duplicate articles were removed. The remaining 206 articles were screened using titles and abstracts. In vitro studies, studies evaluating the efficacy of antibiotics other than systemic amoxicillin–metronidazole, non-randomized controlled trials, case reports and series, and studies evaluating other adjuvants to NSPT, including laser and photodynamic therapy were excluded. The full texts of twelve articles were selected for further review. Based on the predefined eligibility criteria, eight articles were excluded. Two of the articles excluded repeated trial data and were longer-term follow-ups [26,27]. Four articles were selected for inclusion in this review. Figure 1 depicts the PRISMA flow diagram.

### 3.1. Characteristics of the Selected Studies

#### 3.1.1. Characteristics of Study Settings

All four studies included in this review were randomized clinical trials. Two of the studies were conducted in Brazil [28,29], and the other two were conducted in Egypt [30] and Mexico [31]. A summary of the characteristics of the selected studies is shown in Table 1 and Supplementary Table S2.

#### 3.1.2. Characteristics of Interventions

Out of the four studies, three studies evaluated the efficacy of amoxicillin–metronidazole as an adjunct to nonsurgical periodontal therapy [29,30,31], while one of the studies compared amoxicillin–metronidazole as an adjunct to surgical therapy vs. nonsurgical therapy [28]. Miranda et al. [29] and El Makaky et al. [30] used 500 mg amoxicillin and 400 mg metronidazole TID for 14 days. Gomez-Sandoval et al. [31] used 500 mg amoxicillin and 250 mg metronidazole TID for 7 days, and Mendonca et al. [28] used 500 mg amoxicillin and 400 mg metronidazole TID for 10 days.

Two studies reported adverse events, such as diarrhea, headache, and metallic taste [28,29]. However, no statistically significant differences were reported between the treatment groups in either study. Two studies did not report any adverse effects [30,31].

#### 3.1.3. Characteristics of Outcome Measures

All four studies evaluated probing depth (PD) as part of their primary outcomes, along with clinical attachment level (CAL) and bleeding on probing (BOP) [28,29,30,31]. A few studies measured additional parameters, such as HbA1c levels and plaque indexes [30]. Miranda et al. evaluated subgingival microflora along with serum HbA1c levels [24,26,29,31]. Mendonca et al. measured number of residual pockets and cytokine levels in saliva [28].

The included studies measured PD, CAL, and BOP using a manual periodontal probe (North Carolina, Hu-Friedy, Chicago, IL, USA). PD and CAL were measured for six sites around all teeth, excluding third molars. BOP was dichotomously recorded as yes or no based on whether bleeding was present or absent for all the teeth, excluding third molars.

#### 3.1.4. Characteristics of Outcomes

All four studies showed that systemic amoxicillin–metronidazole improved periodontal health when used in conjunction with nonsurgical periodontal therapy. Miranda et al. and El Makaky et al. demonstrated a greater reduction in PD, CAL, and BOP at 3 months when systemic amoxicillin–metronidazole was used compared to delayed treatment [30] or SRP alone [29]. Miranda et al. also assessed the microbial species cultured from subgingival biofilm and observed reductions in numbers of *T. denticola*, *P. gingivalis*, *T. forsythia*, *Eubacterium nodatum*, and *Prevotella intermedia* in the test group when compared to the control group [29].

Gomez-Sandoval et al. compared systemic amoxicillin–metronidazole with systemic clindamycin–placebo and observed no statistically significant differences between either group regarding a reduction in pocket probing depth and other clinical parameters, such as plaque index and bleeding on probing [31].

Mendonca et al. compared nonsurgical therapy with surgical therapy in conjunction with systemic amoxicillin–metronidazole. The authors observed that while both treatment groups showed a reduction in PD and number of residual pockets, the reduction was more significant in the surgical group [28].

### 3.2. Quality of the Evidence

**Figure 2:**
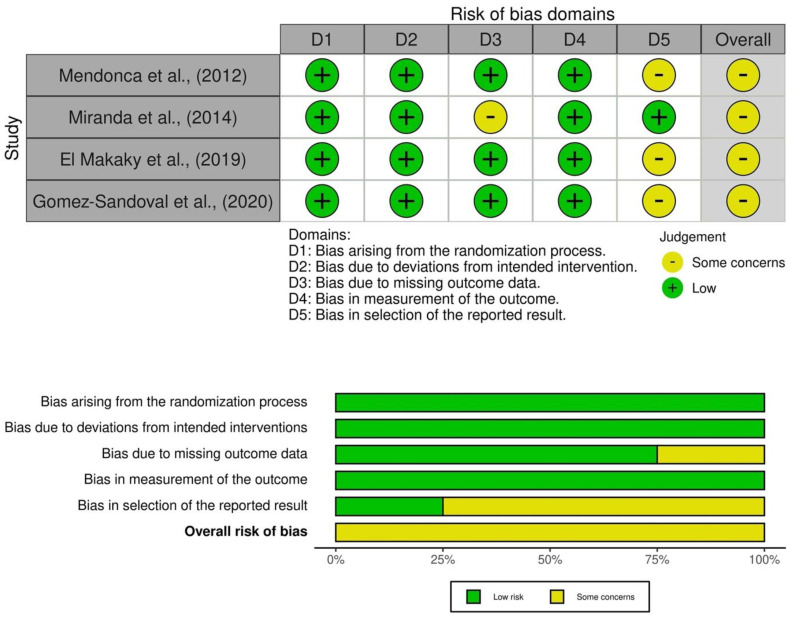
Summary of the risk of bias [28,29,30,31].

Based on ROB2, all included trials presented some concerns based on methodological insufficiencies [28,29,30,31]. These were mainly related to dropouts resulting in missing outcome data and concerns regarding selective reporting. Figure 2 provides a summary of the risk of bias [32].

A total of 209 patients were involved in this review. The overall quality of evidence was assessed as low according to GRADE. Two of the studies produced a null effect for the primary outcome parameter and had imprecision in reporting [28,31]. The risk of bias was categorized as “some concerns” due to missing information regarding the blinding of the investigators. Table 2 provides a summary of findings using the GRADE system.

## 4. Discussion

Nonsurgical periodontal therapy (NSPT), including scaling and root planing, combined with patient education and motivation was proven to be effective in reducing clinical and inflammatory parameters in patients with poorly controlled diabetes. However, evidence on the adjunctive use of antibiotics, antimicrobials, or antiseptics to NSPT is scarce [14,15]. This systematic review assessed the efficacy of systemic amoxicillin in combination with metronidazole in managing patients with diabetes presenting with diabetes mellitus. Although the initial search yielded 611 articles, only 4 were found to adhere to the study eligibility criteria completely and to present the results of randomized trials. Three of the included studies examined systemic amoxicillin–metronidazole as an adjunct to scaling and root planing (SRP) compared to no treatment or scaling and root planing (SRP) alone [29,30,31]. One study compared systemic amoxicillin–metronidazole with NSD against systemic amoxicillin–metronidazole with surgical debridement [28]. All four studies examined measured probing depth (PD), clinical attachment level (CAL), and bleeding on probing (BOP) as outcomes.

All four studies showed that systemic amoxicillin–metronidazole with nonsurgical periodontal therapy can improve periodontal health. Two of the studies showed reductions in PD, CAL, and BOP compared to SRP or delayed treatment alone [29,30]. A reduction in red-complex species was also observed by Miranda et al. Two-year and five-year follow-ups to this trial were published by Tamashiro et al. [26] and Cruz et al. [27], respectively. At the 2-year follow-up appointment, the lowest mean proportions of red-complex pathogens were associated with the test group. Test-group patients also presented with fewer sites of BOP and suppuration, more significant mean PD reductions, and greater CAL gains [26]. However, over the 5-year follow-up, since no supportive periodontal therapy was performed in between, some of the clinical and microbiological benefits observed at 2 years were lost. Even so, the results were better maintained in the test group, and in the control group the parameters were observed to return to baseline levels [27].

Systemic amoxicillin–metronidazole is associated with improvements in clinical outcomes in systemically healthy patients. Sgolastra et al. observed that systemic amoxicillin–metronidazole, as an adjunct to scaling and root planing (SRP), is associated with better CAL gain and PD reduction. However, no significant effect was found on BOP and suppuration. The authors concluded that amoxicillin–metronidazole, as adjuncts to SRP, effectively managed chronic periodontitis [33]. Souto et al. reviewed eleven trials with different systemic antibiotics, such as doxycycline, amoxicillin with metronidazole, amoxicillin with clavulanic acid, and azithromycin, as adjuncts to nonsurgical therapy in patients with diabetes and periodontitis. Most of the trials assessed the efficacy of doxycycline with nonsurgical therapy. The most significant reduction in probing pocket depth (PPD) was associated with amoxicillin–metronidazole [19]. These findings agree with the results of our review showing that the amoxicillin–metronidazole combination can improve periodontal parameters.

Mendonca et al. determined that, while nonsurgical and surgical periodontal therapy helped reduce PD and CAL, surgical debridement was more effective than nonsurgical therapy, especially at sites with initially deep pockets [28]. The meta-analysis by Hung et al. corroborated these findings in non-diabetic individuals [34]. The authors noted that surgical periodontal therapy could be more effective in reducing PD in initially deeper pockets. However, the attachment gain was similar between surgical and nonsurgical therapy [34].

Although systemic antibiotics are prescribed to reduce periodontopathogenic microflora, since prior antimicrobial testing is not always performed or possible, these treatments have been used empirically, with wide variations in the initiation, dosage, and duration of the prescribed antibiotics [21]. The development of antibiotic resistance and the severe adverse effects reported are primary concerns of medical professionals today and have been associated with significant mortality. Antibiotic stewardship programs have been instituted across various organizations to reduce sub-optimal and sometimes unnecessary usage of antibiotics [35]. Periodontists prefer using an amoxicillin–metronidazole combination due to its broad spectrum of action and efficacy against anaerobic organisms [22]. However, the side effects, such as vomiting, nausea, and metallic taste, associated with metronidazole prevent widespread use of this drug without correct guidelines. The results of the present review demonstrate positive effects associated with systemic amoxicillin–metronidazole as an adjunct to SRP compared to either delayed treatments or SRP alone. However, compared to another systemic antibiotic, clindamycin, amoxicillin–metronidazole failed to demonstrate any significant advantages. When comparing surgical debridement to nonsurgical therapy in conjunction with systemic amoxicillin–metronidazole, surgical therapy proved to be more effective with better longer-term PD and CAL reduction.

### 4.1. Completeness and Applicability of Evidence

All four of the trials included in the study were randomized clinical trials. However, the findings of these trials did not provide a complete picture of the outcomes at all timepoints. The studies used different dosages and durations for the amoxicillin–metronidazole combination treatments. Variations in dosage might impact the effects of interventions and lead to divergences in the effects between studies. The follow-up times varied between the studies. Two studies were limited to less than a year of follow-up [28,30]. Longer follow-up periods are necessary to identify persistent effects that may not be immediately detectable. Two studies did not mention any adverse effects affecting the treatment groups [30,31]. Additionally, adverse effects and patient-reported outcomes may affect compliance. Unexpected and uneasy side effects may also hinder compliance. Although there is evidence that the use of amoxicillin–metronidazole improved periodontal parameters in patients with diabetes, a definitive consensus could not be reached on its efficacy. A meta-analysis of trial data was not possible due to the heterogeneity in the trial design, interventions, and outcomes reported.

### 4.2. Quality of the Evidence

As per the GRADE guidelines [25], the quality of evidence for the primary outcome, a reduction in pocket probing depth, was low. The certainty of evidence was downgraded to low for both primary and secondary outcomes due to limitations in the study design and reporting. Our confidence in the effect estimate was also limited, and the true effect may be different from the estimate. This issue can be principally attributed to the imprecision in data reporting and some concerns in the risk of bias among included studies. Moreover, two studies reported a null effect for the primary outcome, which should be explored in future studies.

The findings of this review must be interpreted with caution due to the low certainty of evidence. Furthermore, these findings have limited generalizability. Periodontal disease and diabetes are associated with reduced renal function [36,37], which may preclude the use of antibiotics in their treatment. Further research will likely have an effect on the findings of our review.

A wide-ranging search strategy was employed to identify studies for inclusion in this review. Multiple authors independently assessed eligibility using well-defined inclusion criteria to minimize any selection bias. Our review was confined to published randomized clinical trials. Moreover, this study was limited by the inclusion of articles published only in the English language due to a lack of translational resources. This factor may have resulted in publication bias. Further long-term randomized controlled trials are required before the systemic amoxicillin–metronidazole combination can be safely and effectively included in good-practice guidelines for managing periodontitis in patients with diabetes. Out of the four included articles, only Miranda et al. provided a long-term (2-year and 5-year) evaluation of outcomes [26,27,29]. At minimum, a 12-month follow-up is recommended, as some studies demonstrated no significant changes with adjuvant systemic antibiotics compared to SRP alone on long-term follow-up.

Comparing traditional drugs, such as amoxicillin–metronidazole, with other systemic medications, such as clindamycin, azithromycin, and doxycycline, can be beneficial in reaching a consensus. It is also critical to evaluate whether systemic antibiotics are superior to local antibiotic therapy in managing periodontitis in patients with diabetes. It might also be interesting to assess the changes in HbA1c levels under different therapeutic modalities.

One of the limitations of the protocol was the decision to exclude studies with missing outcome data that were not considered for statistical analysis and therefore could have biased the trial results. However, none of the studies had to be excluded during the screening due to missing outcome data. Miranda et al. reported patient dropout during the study; however, this was adjusted for statistical analysis, and the trial was included in the systematic review with the risk of bias recorded accordingly [29]. Considering the limited number of trials published evaluating systemic amoxicillin–metronidazole in patients with diabetes, Mendonca et al. provided significant insight into the efficacy of the drug combination when used as an adjunct to surgical and non-surgical periodontal therapy, and this study was included in the present review even though it is not standard protocol to included pilot studies in systematic reviews [28].

## 5. Conclusions

Overall, the current body of evidence suggests that systemic amoxicillin–metronidazole can elicit better clinical outcomes when combined with nonsurgical periodontal therapy. However, evidence from the selected four trials on the efficacy of systemic antibiotics is limited and offers low certainty. Disparities were observed in the treatments performed on the control groups and in the doses and durations of the antibiotic prescriptions. Future research should ensure the uniformity of these parameters and include patient-reported outcomes of discomfort following treatment, along with reports of adverse effects. Longer follow-up times in studies should also be considered. Further research will provide a fuller picture of effective intervention strategies against periodontitis in patients with diabetes.

## Figures and Tables

**Table 1:** Characteristics of the selected studies.

Author	Year	Country	Sample Size	Study Design	Intervention	Control Used	Outcome Assessment	Primary Outcome	Secondary Outcomes	Inference
El-Makaky [30]	2019	Egypt	88 patients (Test group = 44, Control group = 44)	Test group: Full mouth scaling and root planing (SRP) + amoxicillin (500 mg TID for 2 weeks) + metronidazole (400 mg TID for 2 weeks); Control group: No treatment was used during the 3-month follow-up, after which treatment was the same as that used in the test group (delayed treatment)	Amoxicillin (500 mg TID for 2 weeks) + metronidazole (400 mg TID for 2 weeks)	Delayed treatment (Periodontal treatment performed only after 3 months from baseline)	PD, CAL, BOP	Test group—baseline: 4.99 ± 0.75, at 3 months: 4.27 ± 0.88; Control group—baseline: 5.01 ± 0.83, at 3 months: 5.1 ± 0.83	CAL at baseline—test group: 5.27 ± 0.80 and control group: 5.24 ± 0.82; CAL at 3 months—test group: 4.47 ± 0.73 and control group: 5.41 ± 0.81; BOP at baseline—test group: 45.64 ± 28.34 and control group: 46.27 ± 20.73; BOP at 3 months—test group: 15.36 ± 9.82 and control group: 49.23 ± 20.75	The nonsurgical periodontal treatment using a combination of metronidazole and amoxicillin significantly improved the metabolic outcomes in addition to periodontal health in subjects with diabetes with chronic periodontitis
Miranda et al. [29]	2014	Brazil	56 subjects (Test group = 29, Control group = 27)	Test group: SRP + amoxicillin (500 mg TID for 14 days) + metronidazole (400 mg TID for 14 days); Control group: SRP + placebo	Amoxicillin (500 mg TID for 14 days) + metronidazole (400 mg TID for 14 days)	Placebo	PD, CAL, BOP	Test group—baseline: 3.6 ± 0.5, at 3 months: 2.6 ± 0.2; Control group—baseline: 3.6 ± 0.6, at 3 months: 3.0 ± 0.5	CAL at baseline—test group: 4.6 ± 1.2 and control group: 4.6 ± 0.8; CAL at 3 months—test group: 3.8 ± 0.9 and control group: 4.1 ± 0.8; BOP at baseline—test group: 40.7 ± 14.0 and control group: 34.2 ± 15.9; BOP at 3 months—test group: 9.7 ± 4.9 and control group: 17.0 ± 7.9	The adjunctive use of MTZ + AMX significantly improved the clinical and microbiological outcomes of SRP in the treatment of type 2 diabetes subjects with ChP
Gómez-Sandoval JR et al. [31]	2020	Mexico	42 subjects (AMX + MET group = 21, Clindamycin group = 21)	AMX + MET group: Standard periodontal therapy + amoxicillin (500 mg TID for 7 days) + metronidazole (250 mg TID for 7 days); Clindamycin group: Standard periodontal therapy + clindamycin (300 mg TID for 7 days) + placebo (TID for 7 days)	Amoxicillin (500 mg TID for 7 days) + metronidazole (250 mg TID for days)	Clindamycin (300 mg TID for 7 days) + placebo (TID for 7 days)	PD, BOP	Baseline: AMX + MET group: 2.4 ± 0.6; Clindamycin group: 2.6 ± 0.6; 3-month values not reported; comparison between the 2 groups at 24 months gave a *p*-value of 0.624	Baseline BOP—AMX + MET group: 42.4 ± 24.2; Clindamycin group: 55.6 ± 25.8; 3-month values not reported; comparison between the 2 groups at 24 months gave a *p*-value of 0.163	The administration for 7 days of clindamycin or amoxicillin/metronidazole showed the same efficacy in terms of reductions in probing depth, plaque index, and bleeding on probing in patients with periodontitis and type 2 diabetes
Mendonca et al. [28]	2012	Brazil	21 patients (Surgical group: *n* = 21, pockets = 178; Non-surgical debridement: *n* = 21, pockets = 141)	NSD group: SRP + amoxicillin (500 mg TID for 10 days) + metronidazole (400 mg TID for 10 days); SD group: Open flap debridement + amoxicillin (500 mg TID for 10 days) + metronidazole (400 mg TID for 10 days)	SRP + amoxicillin (500 mg TID for 10 days) + metronidazole (400 mg TID for 10 days)	Open flap debridement + amoxicillin (500 mg TID for 10 days) + metronidazole (400 mg TID for 10 days)	PD, CAL, BOP	Baseline: NSD group: 3.8 ± 0.5; SD group: 4.3 ± 1.0; at 3 months: NSD group: 3.1 ± 0.8; SD group: 3.4 ± 0.3	CAL at baseline—NSD group: 4.5 ± 0.9 and SD group: 5.0 ± 1.0; CAL at 3 months—NSD group: 4.3 ± 1.5 and SD group: 4.3 ± 1.2; BOP at baseline—NSD group: 34 ± 17 and SD group: 43 ± 24; BOP at 3 months—NSD group: 16 ± 15 and SD group: 22 ± 22	Even though both groups showed reduced PD and CAL, SD associated with systemic antimicrobials provided a more significant reduction in PD and CAL

**Table 2:** Summary of findings.

Quality Assessment						Summary of Findings		
Outcome	Risk of Bias	Inconsistency	Indirectness	Imprecision	Publication Bias	Impact	Number of Participants (Studies)	Certainty of Evidence (GRADE)
Reduction in periodontal probing depth	Some concerns	Not serious	Not serious	Serious ^a^	Unlikely	Only a weak recommendation can be made	209 (4)	Low
Bleeding on probing and clinical attachment loss	Some concerns	Not serious	Not serious	Serious ^b^	Unlikely	Only a weak recommendation can be made	209 (4)	Low

^a^ Two of the studies showed null effects for outcomes. ^b^ Two of the studies showed null effects for outcomes.

## Data Availability

Date will be shared per reasonable request.

## Supplementary Materials

The following supporting information can be downloaded at: https://www.mdpi.com/article/10.3390/medicina58111605/s1, Table S1: Search strategy; Table S2: Comparison of diagnostic criteria of periodontitis, details of type 2 diabetes mellitus, funding, and additional parameters.
