# Supplementary material for: Efficacy of Systemic Amoxicillin–Metronidazole in Periodontitis Patients with Diabetes Mellitus: A Systematic Review of Randomized Clinical Trials
Source: Medicina (Kaunas). 2022 Nov 7;58(11):1605. doi: 10.3390/medicina58111605 (PMC9695465; doi:10.3390/medicina58111605)
Supplement: Supplementary file 1 [file medicina-58-01605-s001.zip › medicina-1979322-supplementary.pdf]

Supplemental Table S1. Search strategy

Source	Keywords	No. of articles	Date
Pubmed	Search: (((periodontitis) AND (diabetes mellitus)) AND (systemic antibiotics)) AND (amoxicillin) AND (metronidazole) AND (nonsurgical therapy) Filters: Randomized Controlled Trial, from 2000/1/1 - 2022/1/31<br>(("periodontal"[All Fields] OR "periodontally"[All Fields] OR "periodontically"[All Fields] OR "periodontics"[MeSH Terms] OR "periodontics"[All Fields] OR "periodontic"[All Fields] OR "periodontitis"[MeSH Terms] OR "periodontitis"[All Fields] OR "periodontitides"[All Fields]) AND ("diabetes mellitus"[MeSH Terms] OR ("diabetes"[All Fields] AND "mellitus"[All Fields]) OR "diabetes mellitus"[All Fields]) AND (("systemic"[All Fields] OR "systemically"[All Fields] OR "systemics"[All Fields]) AND ("anti bacterial agents"[Pharmacological Action] OR "anti bacterial agents"[MeSH Terms] OR ("anti bacterial"[All Fields] AND "agents"[All Fields]) OR "anti bacterial agents"[All Fields] OR "antibiotic"[All Fields] OR "antibiotics"[All Fields] OR "antibiotic s"[All Fields] OR "antibiotical"[All Fields])) AND ("amoxicillin"[MeSH Terms] OR "amoxicillin"[All Fields] OR "amoxicilline"[All Fields] OR "amoxicillins"[All Fields]) AND ("metronidazole"[MeSH Terms] OR "metronidazole"[All Fields] OR "metronidazol"[All Fields] OR "metronidazoles"[All Fields]) AND ("non-surgical"[All Fields] AND ("therapeutics"[MeSH Terms] OR "therapeutics"[All Fields] OR "therapies"[All Fields] OR "therapy"[MeSH Subheading] OR "therapy"[All Fields] OR "therapy s"[All Fields] OR "therapys"[All Fields]))) AND ((randomizedcontrolledtrial[Filter]) AND (2000/1/1:2022/1/31[pdat]))	20	31.01.2022
WOS	((((TS=(periodontitis)) AND TS=(diabetes mellitus)) AND TS=(systemic antibiotics)) AND TS=(amoxicillin)) AND TS=(metronidazole)) AND TS=(nonsurgical therapy) and Clinical Trial (Document Types) and English (Languages)	24	31.01.2022
Scopus	(ALL (periodontitis) AND ALL (diabetes AND mellitus) AND ALL (systemic AND antibiotics) AND ALL (amoxicillin AND with AND metronidazole) AND ALL (non-surgical AND therapy) AND ALL (randomized AND clinical AND trial)) AND PUBYEAR > 1999 AND PUBYEAR < 2023 AND (LIMIT-TO (DOCTYPE , "ar")) AND (LIMIT-TO (LANGUAGE , "English")) AND (LIMIT-TO (SRCTYPE , "j"))	567	31.01.2022

Supplemental Table S2. Comparison of diagnostic criteria of periodontitis, details of type 2 diabetes mellitus, funding, and additional parameters.

Author	Year	Age	Diagnosis of Periodontitis	Duration of DM	Mean HbA1c	Microbiological Parameters	Cytokine Levels	Follow-Up	Adverse Effects	Funding
El-Makaky	2019	Test group: 52.95±6.523 Control group:52.23 ± 7.028	Chronic periodontitis diagnosed as presence of ≥4 teeth with CAL ≥ 3 mm and PPD ≥ 4 mm in more than 30% of sites	Uncontrolled type 2 diabetes (HbA1c from 7% to 9%) diagnosed for more than 5 years	Test group-baseline: 8.12±0.74 and at 3 months: 7.27±0.5; Control group-baseline: 8.21±0.71 and at 3 months: 8.21±0.71			3 months	No adverse events reported	Funded by authors
Miranda et al	2014	Test group: 54.0 ± 8.2 and Control group:53.7 ± 8.0	Generalized chronic periodontitis - more than 30% of the sites with PD and CAL ≥ 4 mm and ≥ 6 teeth with at least one site with PD and CAL ≥ 5 mm and bleeding on probing (BoP) at baseline	Type 2 DM for ≥ 5 years, treated with diet and insulin supplementation or oral hypoglycemic agents, mean duration of DM- Test group: 8.0 ± 3.2 and Control group: 7.4 ± 3.6	Test group-baseline: 8.53 ± 1.56 and at 3 months: 8.60 ± 2.01; Control group-baseline: 8.99 ± 1.63 and at 3 months: 8.94 ± 1.71	The antibiotic-treated group also presented reduced levels and greater decreases of the three red complex species, Eubacterium nodatum and Prevotella intermedia, compared to the control group at 1 year		12 months with follow-up at 3 months and 6 months	Diarrhea (Test group: n=7;Control group n=3) Headache (Test group: n=4; Control group n=1) Metallic taste (Test group: n=4 ;Control group n=2) Nausea/Vomiting (Test group: n=5;Control group n=2)	São Paulo State Research Foundation (São Paulo, São Paulo, Brazil, # 2011/14872-4; 2013/01072-5)
Gómez-Sandoval JR et al	2020	AMX+MET group: 52.5 ± 8.0 and Clindamycin group: 52.0 ± 10.6	Moderate localized chronic periodontitis	Type 2 diabetes with a HbA1C<8% and a fasting plasmaglucose (FPG) <180 mg/dL diagnosed less than 10 years back	AMX+MET group: 7.1 ± 0.4; Clindamycin group: 7.3 ± 0.4			24 months	No adverse events reported	No specific grant from any funding agency in the public, commercial or not-for-profit sectors
Mendonca et al	2012	53.2 ± 9.1 years	Generalized chronic periodontitis with at least two non-contiguous RP per half contralateral quadrant at 12 months after pre-study periodontal therapy	7.1 ± 0.9 years	11.3 ± 2.3%		Levels of all cytokines - IL-4, IFN-γ, IL-17 and IL-23 were increased after SD compared with NSD	6 months with 3 month follow-up	diarrhoea, vomiting, headache, metallic taste or irritability	São Paulo State Research Foundation (São Paulo, São Paulo, Brazil, #2008/09687-0; #2008/04280-0) and Maranhão State Research Foundation (São Luı´s, Maranhão, Brazil)
